# Impact of maternal antepartum depressive and anxiety symptoms on birth outcomes and mode of delivery: a prospective cohort study in east and west coasts of Malaysia

**DOI:** 10.1186/s12884-019-2349-9

**Published:** 2019-06-14

**Authors:** Hashima E. Nasreen, Hafizah Binti Pasi, Sakinah Md Rifin, Mohd Aznan Md Aris, Jamalludin Ab Rahman, Razman Mohd Rus, Maigun Edhborg

**Affiliations:** 1 Department of Community Medicine, Faculty of Medicine, International Islamic University Malaysia, Jalan Sultan Ahmad Shah, 25200 Kuantan, Pahang Malaysia; 2 Department of Family Medicine, Faculty of Medicine, International Islamic University Malaysia, Jalan Sultan Ahmad Shah, 25200 Kuantan, Pahang Malaysia; 3 Department of Neurobiology, Care Sciences and Society, Karolinska Institute, SE-141 83 Huddinge, Stockholm, Sweden

**Keywords:** Antepartum depressive symptoms, Antepartum anxiety symptoms, Low birth weight, Preterm birth, Caesarean section, Instrumental delivery, Malaysia

## Abstract

**Background:**

Antepartum depressive and anxiety symptoms (ADS and AAS) are prevalent in Malaysia. Prior evidence linking maternal ADS and AAS with adverse birth outcomes and caesarean section (CS) or instrumental delivery is conflicting. There is no research in Malaysia on the association between maternal mental disorders and adverse birth outcomes and mode of delivery. This study aims to investigate the independent effect of maternal ADS and AAS on low birth weight (LBW), preterm birth (PTB) and CS or instrumental delivery among women in east and west coasts of Malaysia.

**Methods:**

We used data from a prospective cohort study of 799 pregnant women from health clinics of two states in east and west coasts of Malaysia. Baseline data were measured at the third trimester of pregnancy on ADS, AAS, socioeconomic condition, anthropometric status, reproductive history and intimate partner violence. Birth outcomes and mode of delivery were determined at the time of delivery. Univariate and multiple Cox’s regressions were applied to assess the association between ADS and AAS and LBW, PTB and CS or instrumental delivery.

**Results:**

ADS was significantly associated with an increased risk of giving birth to LBW babies in both east coast (RR = 3.64; 95% CI 1.79–7.40) and west coast (RR = 3.82; 95% CI 1.86–7.84), but not with PTB. AAS was associated with increased risk of both LBW (RR = 2.47; 95% CI 1.39–4.38) and PTB (RR = 2.49; 95% CI 1.16–5.36) in the east coast, but not in west coast. The risk of CS or instrumental delivery was evident among women with ADS (RR = 2.44; 95% CI 1.48–4.03) in west coast only.

**Conclusion:**

ADS predicts LBW in both coasts, AAS predicts LBW and PTB in east coast, and ADS predicts CS or instrumental delivery in west coast. Policies aimed at detection and management of ADS and AAS during antenatal check-up in health clinics may help improve birth outcomes and reduce obstetric interventions.

## Background

Depression and anxiety are common complications during pregnancy and childbearing age [1], and are recognized as factors that may adversely impact on maternal and neonatal outcomes [2]. The prevalence of antepartum depressive symptoms (ADS) is estimated at 15.6% in low and lower middle income countries [3], which is higher than that reported from high income countries (12%) [4]. The pooled prevalence is 15.2% for any antepartum anxiety disorder and 22.9% for antepartum anxiety symptoms (AAS) [5]. Experiencing ADS or AAS may expose both mothers and infants to psychological risks, such as impaired bonding to the foetus [6] and physiological consequences including low intra-uterine growth, low birth weight (LBW; less than 2500 g) and preterm birth (PTB; birth before 37 weeks of gestation) [7–10]. ADS or AAS may also affect the mode of delivery [11] and women’s preference for a caesarean section (CS) [12]. LBW and PTB are the leading causes of neonatal and infant morbidity, mortality [13], and neurodevelopmental impairments and disabilities [14]. Globally, it is estimated that nearly 22 million newborns, accounting for 16% of all births, were born with LBW. The incidence of PTB has been estimated to be 9.6% of all births, which corresponds to 12.9 million PTB worldwide [15]. Approximately 85% of these burdens concentrate in Africa and Asia [16]. There are a number of well-known risk factors for LBW, PTB and CS delivery including preeclampsia, hypertension, gestational diabetes, and intimate partner violence (IPV) [7, 17, 18].

Several systematic reviews and meta-analyses have been carried out showing inconsistency and inconclusive association among ADS, PTB and LBW [7–10]. From a meta-analyses of 29 studies Grote et al. [7] reported significant associations among ADS, LBW and PTB. Accortt et al. [9] included 95 studies between 1977 and 2013 in an updated systematic review and concluded that ADS was rarely associated with PTB in larger, well-controlled studies. Another systematic review and meta-analysis about ADS and adverse birth outcomes found PTB significantly associated with ADS, but not with LBW [8].

Since researchers focused more attention on ADS, less studies were done on AAS and birth outcomes. But the high level of comorbidity between ADS and AAS, estimated at 60% [4], suggests anxiety and depression should be examined concurrently. One systematic review explored the effect of antenatal psychological distress on PTB in 39 studies and included 14 studies examining the effect of ADS, four studies examining AAS, and five studies stress [1]. The findings suggest an increased risk of PTB when a woman experiences one or more of the described psychological disorders. However, Grigoriadis et al. [19] found in a recent systematic review and meta-analysis including 29 articles that AAS was associated with both PTB and LBW.

Data on the effect of depression and anxiety on mode of delivery are more limited [12]. Moreover, making an evidence-based decision has been challenging, particularly because of mixed and contradictory findings [20, 21]. In a systematic review of four studies exploring CS following exposure to AAS, three studies showed non-significant results and one significant [19]. However, Bayrampour et al. [11] found that ADS in the third trimester of pregnancy increased the risk of emergency CS, but not AAS.

Given the inconsistent results vis-à-vis the impact of ADS and AAS on adverse birth outcomes and mode of delivery across countries, and particularly less research on AAS, more evidence should be warranted to explore these associations in different settings to inform contextualized interventions. In Malaysia, there are no studies assessing the association among ADS, AAS, LBW, PTB and mode of delivery, where the incidences of LBW and PTB were 11.0 and 11.3%, respectively in 2012 [15], and increased trend of CS delivery from 10.5% in 2000 to 15.7% in 2006 [22]. This prospective cohort study addressed this shortcoming by examining the impact of ADS and AAS on LBW, PTB and CS or instrumental delivery among women in east and west coasts of Malaysia.

## Methods

### Study design and setting

Data for this prospective cohort study were collected at the third trimester of pregnancy and at birth from a larger longitudinal study of perinatal depressive and anxiety symptoms among women in Malaysia. The women were recruited from health clinics in Pahang and Selangor states in the east and west coasts of peninsular Malaysia, respectively [23]. Pahang is predominated by the indigenous and rural culture, which has a population about 1.5 million. The economy in Pahang is primarily based on agriculture and mining, contributing 4.2% to country’s gross domestic product (GDP). The majority of women are involved in unpaid domestic work. Conversely, Selangor is more developed, predominantly urban and contemporary. It has a population of about 5.5 million and is the leading GDP contributor (22.6%) to national economy, where the major sources of economy are commerce, industry and service sectors [24]. Pregnant women in Malaysia get free antenatal and postnatal care at the government health clinics and hospitals, and majority of the births occur at hospitals [23].

### Participants and sample size

**Fig. 1:**
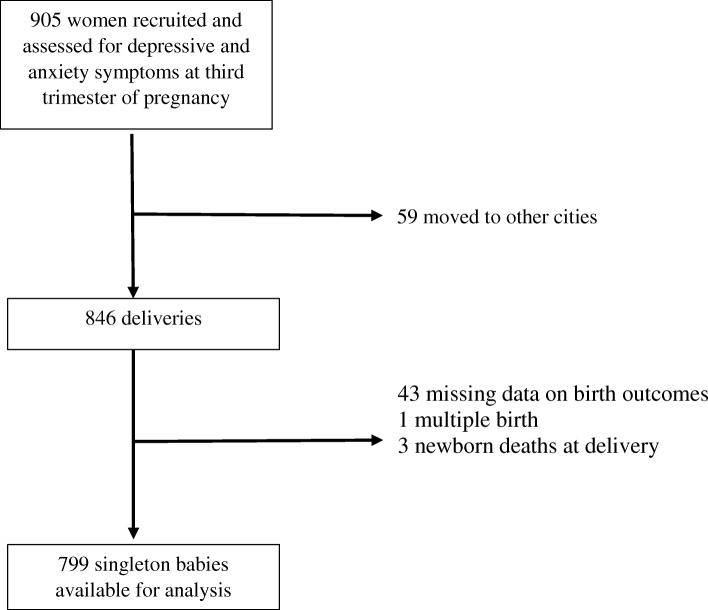
Study participants

Participants were pregnant women enrolled in this study at their third trimester of pregnancy (≥28 weeks) from 10 health clinics where the highest number of attendance for antenatal check-up was observed. Estimation of gestational weeks at enrolment was based on ultrasound scanning during antepartum check-up. A cohort of 905 pregnant women were recruited for the study, who were followed-up until delivery, and delivery information was collected from 799 (88.3%) singleton live births (Fig. 1). The exclusion criteria for the study were non-Malaysian, illiterate, moved out from the study area, multiple birth (as it affects birth outcomes) and intrauterine death. The details about sample size for the study were explained elsewhere [23].

### Data collection

Data for the larger longitudinal study were collected from March 2016 to August 2017. Baseline data were collected at third trimester of pregnancy on socioeconomic and anthropometric status, reproductive history, perceived social support, IPV, depression in earlier pregnancy, and depressive and anxiety symptoms. We collected baseline data through self-reported structured questionnaires in Malay during women’s antenatal care visits at the health clinics. Obstetric data were collected on newborn’s sex, gestational age at delivery, mode of delivery, complications during delivery, live or still birth, birth weight and height from the child health cards of respective health clinics. In Malaysia, all obstetric information including anthropometric measurements of newborn babies are measured within 2–48 h after delivery by the attending health personnel of hospitals where the deliveries took place. All these data and their subsequent growth charts are then recorded in the child health cards of the respective health clinics. Trained nurses distributed the baseline questionnaires to the participating women and gave them information about the study if needed. The nurses and research assistants were trained on the questionnaire and data collection, and they scrutinized the completed questionnaires on the spot for any missing data. The baseline questionnaires were pretested among pregnant women during their antenatal care visits at the International Islamic University Malaysia’s family health clinic and finalized based on feedback received in the field test.

### Measures

The outcome variables used in this study include LBW, PTB and SC or instrumental delivery. LBW was classified according to the World Health Organization definition as newborn babies with a birth weight of < 2500 g and PTB was defined as babies born before 37 weeks of gestation [13]. Mode of delivery was categorized as spontaneous vaginal and CS or instrumental delivery encompassing forceps delivery or vacuum extraction.

The Edinburgh Postnatal Depression Scale (EPDS) was used to assess maternal depressive symptom [25]. The EPDS is a 10 items questionnaire, rated 0–3 on each item and ranging from 0 to 30. The scale rates the intensity of depressive symptoms during the previous 7 days, a higher score indicates more depressive symptoms. The items assessed dysphoric mood (five items), anxiety (two items), guilt (one item), ability to cope with everyday life (one item), and suicidal thought (one item). The EPDS is validated in Malay, reporting sensitivity to be 72.7%, specificity 95%, and positive predictive value 80%, using 11.5 as the cut-off score [26]. Thus, the cut-off score ≥ 12 was used to categorise pregnant women with depressive symptoms in this study. The Cronbach’s alpha of the scale was 0.75 at third trimester of pregnancy.

The anxiety sub-scale of validated Malay version of Depression, Anxiety and Stress Scale (DASS 21) was used to measure maternal anxiety symptoms over the previous week [27]. DASS 21 comprises of 21 questions, subdivided into three domains: depression, anxiety and stress, with seven questions in each domain. The choice of response varied from 0 (‘did not apply at all’) to 3 (‘applied very much’ or ‘most of the time’). In this research we used only the seven anxiety items. As we used the short version of DASS with 21 items, instead of 42, the score on the DASS 21 anxiety scale was multiplied by 2 to calculate final score of anxiety symptoms. The cut-off point ≥8 was used in the study to estimate the prevalence of AAS [28]. The DASS 21 anxiety scale showed a relatively good reliability with Cronbach’s alpha of 0.74.

Socio-demographic data include women’s age, education (primary, secondary or tertiary), occupation (homemaker or employed encompassing government employee, non-government employee and self-employed), and monthly household income (>RM 5599 as high income, RM 2300–5599 as middle income or < RM 2300 as low income) [24]. Parity (primi or multipara) and newborn baby’s sex (girl or boy) were also recorded. Body mass index (BMI) was defined as women’s weight in kilogram divided by the square of height in meter and was categorized as underweight (< 18.5 kg/m^2^), normal (18.5–24.9 kg/m^2^) or overweight/obese (≥25.0 kg/m^2^) [29]. The validated Malay version of Multidimensional Scale of Perceived Social Support (MSPSS) was used to measure social support [30]. MSPSS consists of 12 items, scored on a 7-point scale from 1 (very strongly disagree) to 7 (very strongly agree), with a total score ranging from 1 to 84, a higher score indicates more support. MSPSS showed good internal consistency in this study with the Cronbach’s alpha of 0.93.

IPV was defined by a lifetime experience of physical abuse ever and physical abuse during pregnancy by husbands. Physical abuse includes four items, scored yes (1) and no (0): 1) slapped or thrown object at her, 2) pushed or shoved to the ground, 3) punched or hit, 4) kicked or dragged on the ground [31]. The total score of physical abuse ranged from 0 to 4 and categorized as no act of physical abuse (0) and acts of physical abuse (1–4).

### Statistical analysis

We compared the respondents’ baseline characteristics by sites using independent sample *t* test, chi-square or Fisher’s exact test. Prevalence of ADS and AAS, and incidence of LBW, PTB and CS or instrumental delivery were calculated. Univariate Cox’s regression analyses were carried out to identify possible risk factors of LBW, PTB and CS or instrumental delivery with *p* < 0.05. To control the confounding effects of these possible risk factors on the association between ADS and AAS, and LBW, PTB and CS or instrumental delivery, adjusted risk ratios (aRR) were computed using multiple Cox’s regression analyses. Statistical significance of the RR was tested by confidence interval (CI) at 95%. Because of the main exposure variables, ADS and AAS were entered in all models regardless of univariate statistical significance level. Separate multiple Cox’s regression analyses were performed to show the role of ADS (model I) and AAS (model II) as risk factors of LBW, PTB and CS or instrumental delivery. To examine the moderating effects of site (east coast vs. west coast), stratified analyses were conducted. Any violation of assumptions was observed by examining the collinearity between explanatory variables and outliers in the models.

## Results

The final sample included 799 pregnant women (55.8% form east coast and 44.2% from west coast) with mean age of 29.7 (± 4.7) years in the third trimester of pregnancy. Women in west coast were more educated and employed than women in east coast. Approximately 51% of the participants were from middle income level with median monthly household income of RM 3500 and 21% from high income level with significantly higher proportion fitted in west coast. The average BMI of pregnant women was 27.4 (±5.7) kg/m^2^ and 36% were primiparas. Twenty-one (2.6%) and 13 (1.6%) pregnant women reported of being the victim of at least one act of physical abuse ever and physical abuse during current pregnancy, respectively. There were 12.3% women suffering depressive symptoms and 28.7% anxiety symptoms during pregnancy. The prevalence of AAS was found to be higher in west coast than in east coast (35.1% vs. 23.5%, respectively) (Table 1).

**Table 1:** Baseline characteristics of women at third trimester of pregnancy (in percent unless otherwise stated)

	East coast	West coast		*p* value
	*N* = 446	N^a^	*N* = 353	
Age, mean (SD)	29.8 (5.1)	350	29.6 (4.1)	0.525
Education		352		
Primary	4.0		0.6	< 0.001
Secondary	51.1		32.1	
Tertiary	44.8		67.3	
Occupation		353		
Homemaker	49.1		35.7	< 0.001
Employed	50.9		64.3	
Monthly household income		353		
Low income	36.3		17.8	< 0.001
Middle income	48.0		54.4	
High income	15.7		27.8	
BMI		353		
Underweight	6.3		4.2	0.431
Normal weight	29.8		31.4	
Overweight/obesity	63.9		64.3	
Parity		353		
Primipara	31.4		37.1	0.090
Multipara	68.6		62.9	
Intimate partner violence				
Physical abuse ever	2.5	351	2.8	0.738
Physical abuse during pregnancy	1.6	352	1.7	0.881
Social support, mean (SD)	65.6 (10.3)	353	67.8 (10.2)	0.003
Depression in earlier pregnancy	6.7	350	14.0	0.001
Antepartum depressive symptoms	11.2	353	13.6	0.307
Antepartum anxiety symptoms	23.5	353	35.1	< 0.001

^a^Data available for analysis

The average birth weight of a newborn baby was 3.0 (±0.5) kg and the gestational age at delivery was 39.1 (±1.5) weeks. The percentage of pregnant women having LBW and PTB were 12.6% (*n* = 93) and 6.1% (*n* = 49), respectively. A total of 26.5% (*n* = 212) of our sample had either CS or instrumental delivery. However, no significant differences were noted between sites in the incidence of LBW (11.7% in east coast vs. 11.6% in west coast, *p* = 0.984), PTB (6.5% in east coast vs. 5.7% in west coast, *p* = 0.625) and CS or instrumental delivery (26.5% in east coast vs. 26.6% in west coast, *p* = 0.957). Of the 26 (3.3%) women experienced complications during delivery, 84.6% had CS or instrumental delivery. Complications during delivery was found to be higher in women with ADS (8.2%, *p* = 0.003) and AAS (5.7%, *p* = 0.014) than women without ADS (2.6%) and AAS (2.3%). Depressed women experienced foetal distress and prolonged second stage of labour, while anxious women experienced foetal distress, prolonged second stage of labour, oligohydramnios or type 2 diabetes (not shown).

Univariate regression analyses show that ADS is positively associated with LBW, PTB and CS or instrumental delivery, and AAS with LBW and PTB but not with CS or instrumental delivery. Participants from low income households, who were underweight, who experienced physical abuse ever and/or during pregnancy reported increased risk of LBW and PTB. Primipara women were more likely to give birth to LBW babies and to have CS or instrumental delivery. Women who had social support reported a decreased risk of PTB. Meanwhile, higher BMI of pregnant women, complications during delivery and gestational age were significant predictors for CS or instrumental delivery as compared to others (Table 2).

**Table 2:** Univariate analyses between potential confounders and outcomes under study (*N* = 799)

	Low birth weight		Preterm birth		CS or instrumental delivery	
	n (%)	RR (95% CI)	n (%)	RR (95% CI)	n (%)	RR (95% CI)
Age, mean (SD)	28.5 (4.7)	0.94 (0.89–0.98	29.2 (4.6)	0.97 (0.91–1.03)	30.2 (4.8)	1.02 (0.99–1.05)
Educational level						
Primary/secondary	45 (12.5)	1	26 (7.2)	1	100 (27.7)	1
Tertiary	48 (11.0)	0.76 (0.52–1.18)	23 (5.3)	0.66 (0.38–1.17)	112 (25.6)	0.85 (0.65–1.11)
Occupation						
Home maker	46 (13.3)	1	29 (8.4)	1	89 (25.8)	1
Employed	47 (10.4)	0.72 (0.48–1.08)	20 (4.4)	0.49 (0.28–0.88)	123 (27.1)	0.98 (0.74–1.28)
Monthly household income						
High income	10 (6.0)	1	5 (3.0)	1	52 (31.0)	1
Middle income	42 (10.3)	1.86 (0.93–3.71)	22 (5.4)	1.92 (0.73–5.07)	95 (23.4)	0.79 (0.56–1.11)
Low income	41 (18.2)	3.91 (1.95–7.84)	22 (9.8)	4.02 (1.51–10.68)	65 (28.9)	1.16 (0.80–1.67)
BMI						
Overweight/obesity	38 (7.4)	1	24 (4.7)	1	–	–
Normal weight	29 (11.9)	1.40 (0.86–2.27)	13 (5.3)	1.02 (0.52 2.00)		
Underweight	26 (60.5)	10.50 (6.36–17.32)	12 (27.9)	7.24 (3.61–14.49)		
BMI mean (SD)					28.2 (6.0)	1.04 (1.01–1.06)
Social support, mean (SD)	65.4 (11.6)	0.99 (0.97–1.01)	63.6 (13.7)	0.97 (0.95–0.99)	66.1 (9.9)	0.99 (0.98–1.01)
Parity						
Multipara	42 (8.0)	1	28 (5.3)	1	128	1
Primipara	51 (18.8)	2.42 (1.61–3.65)	21 (7.7)	0.85 (0.84–2.63)	(24.2) 84 (31.0)	1.33 (1.01–1.75)
Physical abuse ever						
No	79 (10.2)	1	41 (5.3)	1	206 (26.5)	1
Yes	14 (66.7)	8.39 (4.73–14.89)	8 (38.1)	9.71 (4.53–20.81)	6 (28.6)	1.43 (0.63–3.22)
Physical abuse during pregnancy						
No	88 (11.2)	1	46 (5.9)	1	208 (26.5)	1
Yes	5 (38.5)	2.96 (1.20–7.33)	3 (23.1)	3.67 (1.13–11.87)	4 (30.8)	0.98 (0.36–2.64)
Depression in earlier pregnancy						
No	76 (10.6)	1	41 (5.7)	1	192 (26.8)	1
Yes	16 (20.3)	1.99 (1.16–3.42)	8 (10.1)	1.85 (0.86–3.95)	19 (24.1)	0.97 (0.61–1.56)
Antepartum depressive symptoms						
No	55 (7.8)	1	32 (4.6)	1	177 (25.2)	1
Yes	38 (38.8)	6.43 (4.21–9.82)	17 (17.3)	4.65 (2.56–8.44)	35 (35.7)	1.80 (1.25–2.59)
Antepartum anxiety symptoms						
No	51 (8.9)	1	28 (4.9)	1	136 (23.9)	1
Yes	42 (18.3)	1.90 (1.26–2.85)	21 (9.2)	1.77 (1.01–3.11)	76 (33.2)	1.29 (0.97–1.70)
Newborn’s sex						
Girl	38 (10.1)	1	18 (4.8)	1	97 (25.9)	1
Boy	55 (13.0)	1.23 (0.85–1.95)	31 (7.3)	1.51 (0.85–2.69)	115 (27.1)	1.05 (0.80–1.38)
Complications during delivery						
No	89 (11.5)	1	47 (6.1)	1	190 (24.6)	1
Yes	4 (15.4)	1.45 (0.53–3.94)	2 (7.7)	1.34 (0.33–5.52)	22 (84.6)	3.86 (2.48–6.01)
Birth weight, mean (SD)	–	–	–	–	3.0 (0.5)	0.89 (0.66–1.20)
Gestational week, mean (SD)	–	–	–	–	39.9 (1.5)	0.85 (0.78–0.92)

Tables 3, 4 and 5 show the associations between ADS and AAS and LBW, PTB and CS or instrumental delivery. After adjusted with all factors that were significant in the crude analysis, pregnant women with ADS had an increased risk of giving birth to babies with LBW (RR 3.58; 95% CI 2.16–5.94) as compared to women without depressive symptoms in the final model, but women with AAS did not. Further stratified analyses show that the association between ADS and LBW remained with the modest changes in risks in both east coast (RR 3.64; 95% CI 1.79–7.40) and west coast (RR 3.82; 95% CI 1.86–7.84). However, AAS had emerged as a risk factor for LBW in east coast (RR 2.47; 95% CI 1.39–4.38) and no association between ADS and LBW existed in west coast. Women’s older age, low and middle income, primipara, women’s underweight and physical abuse ever were also associated with LBW in west coast, and only women’s underweight in east coast (Table 3).

**Table 3:** Association between antepartum depressive and anxiety symptoms and LBW

	Final model	Stratified model	
	aRR (95% CI)	East coast aRR (95% CI)	West coast aRR (95% CI)
Model I			
Women’s age	–	–	1.12 (1.01–1.24)
Middle income	–	–	5.07 (1.65–15.59)
Low income	2.18 (1.02–4.67)	–	5.01 (1.25–20.15)
Primipara	2.46 (1.62–3.71)	2.00 (1.23–3.54)	5.17 (2.29–11.72)
Underweight	3.82 (2.07–7.03)	4.69 (2.24–9.79)	6.12 (1.69–28.05)
Physical abuse ever	2.41 (1.22–4.74)	–	7.18 (2.00–18.64)
Antepartum depressive symptoms	3.58 (2.16–5.94)	3.64 (1.79–7.40)	3.82 (1.86–7.84)
Model II			
Women’s age	–	–	1.12 (1.01–1.24)
Middle income	–	–	4.61 (1.57–13.56)
Low income	2.48 (1.18–5.20)	–	5.82 (1.61–21.09)
Primipara	2.35 (1.55–3.56)	–	5.75 (2.59–12.77)
Underweight	5.48 (3.03–9.78)	7.92 (4.17–15.04)	7.84 (2.71–22.66)
Physical abuse ever	4.52 (2.36–8.66)	–	14.76 (5.63–38.67)
Antepartum anxiety symptoms	–	2.47 (1.39–4.38)	–

**Table 4:** Association between antepartum depressive and anxiety symptoms and PTB

	Final model	Stratified model	
	aRR (95% CI)	East coast aRR (95% CI)	West coast aRR (95% CI)
Model I			
Underweight	3..61 (1.60–8.14)	9.53 (4.31–21.05)	–
Physical abuse ever	3.50 (1.41–8.67)	–	6.63 (1.92–22.91)
Antepartum depressive symptoms	2.36 (1.12–4.99)	–	–
Model II			
Underweight	4.97 (2.33–10.63)	7.39 (3.25–16.78)	–
Physical abuse ever	5.24 (2.25–12.21)	–	6.63 (1.92–22.91)
Antepartum anxiety symptoms	–	2.49 (1.16–5.36)	–

**Table 5:** Association between antepartum depressive and anxiety symptoms and caesarean section or instrumental delivery

	Final model	Stratified model	
	aRR (95% CI)	East coast aRR (95% CI)	West coast aRR (95% CI)
Model I			
Primipara	1.34 (1.02–1.76)	1.47 (1.01–2.15)	–
Women’s BMI	1.04 (1.02–1.06)	1.03 (1.01–1.06)	–
Gestational age	0.86 (0.79–0.93)	0.79 (0.71–0.87)	–
Complications during delivery	3.29 (2.09–5.20)	6.10 (3.30–11.26)	2.05 (1.03–4.08)
Antepartum depressive symptoms	1.55 (1.06–2.26)	–	2.44 (1.48–4.03)
Model II			
Primipara	1.33 (1.01–1.75)	1.47 (1.01–2.15)	–
Women’s BMI	1.04 (1.02–1.06)	1.03 (1.01–1.06)	–
Gestational age	0.86 (0.79–0.93)	0.79 (0.71–0.87)	–
Complications during delivery	3.73 (2.40–5.81)	6.10 (3.30–11.26)	2.84 (1.47–5.47)
Antepartum anxiety symptoms	–	–	–

In the final model, ADS was independently associated with PTB (RR 2.36; 95% CI 1.12–4.99), but AAS was not (Table 4). Conversely, after stratification, ADS was no longer associated with PTB, neither in east coast nor in west coast. However, AAS was found to be an independent risk factor for PTB in east coast (RR 2.49; 95% CI 1.16–5.36) together with women’s underweight. Physical abuse ever was the only risk factor for PTB in west coast (Table 4).

With respect to the mode of delivery, the final model reveals that women with ADS had 55% increased risk for giving birth through CS or instruments. In the stratified analyses, a stronger RR was found in the association between ADS and giving birth through CS or instruments in west coast, but not in east coast. AAS was not associated with increased risk of giving birth through CS or instruments, neither in final model nor in stratified model. Complications during delivery was a strong risk factor for CS or instrumental delivery in both sites. The risk of CS or instrumental delivery increased with primipara and women’s higher BMI, and decreased with higher gestational age in east coast (Table 5).

## Discussion

In this study we analysed the associations between depressive and anxiety symptoms in the third trimester of pregnancy and LBW, PTB and CS or instrumental delivery in a cohort of Malaysian women in east and west coasts. We found that of the newborns 12.6% were born with LBW and 6.1% were PTB. ADS was an independent risk factor for LBW in the final model as well as in both coasts. According to PTB, the significant association was observed in the final model, but not in stratified models, neither in the east nor in the west coast. This association is independent of the effect of maternal nutritional and socioeconomic status (SES). Our results are in agreement with a meta-analysis, where Grote et al. [7] found a strong association between ADS and LBW, and that the risk was higher in low and middle income than in high income countries. Accortt et al. [9] also confirmed our results in their systematic review and reported that more studies (53%) showed significant associations between ADS and LBW than between ADS and PTB (23%). They concluded that ADS appeared to be a greater risk factor for LBW than PTB. Studies from Bangladesh [32] and South Africa [18] reported similarly that ADS was more consistently predicted LBW, but not PTB. However, our results are contrasted by, for example, Grigoriadis et al. [8], Jarde et al. [10] and Staneva et al. [1], who found no significant association between ADS and LBW, instead they found ADS associated with PTB. Nevertheless, it is difficult to determine whether there is an etiological heterogeneity across these settings, because of the different cultures, healthcare systems, and maternal and child health profile.

According to AAS, we found no significant association with LBW and PTB in the final models. But moderator analyses reveal that AAS had increased risks for both LBW and PTB in the east coast, but not in the west coast. Although AAS is reported to be more common in women with lower SES than in women with higher SES [7], more women in the west coast (35.1%) reported AAS compared to women in the east coast (23.5%). Despite, it was women with AAS and underweight in the east coast with lower SES compared to most prosperous women in west coast, who were more likely to give birth to LBW babies and PTB. This is consistent with a systematic review, where Ding et al. [33] reported that AAS was associated with increased risk of PTB and LBW, particularly among Asian women with low SES. Women’s lower SES i.e. less education associated with less employment and low income may deleteriously affects women’s health behaviour and mental state. Our results are consistent with a systematic review and meta-analysis [19] arguing the strength of relationship between AAS and LBW and PTB, hence replicating the results from Asian countries. Consistent with other research [19], our findings suggest that AAS (28.7%) is more prevalent than ADS (12.3%), it appears that the consequences of this disorder may be more adverse as well. Yang et al. [34] reported that only ADS or only AAS was not associated with LBW and PTB among Chinese women, but if participants had LBW with PTB and ADS and AAS co-occur, there was an increased risk. Therefore, public health efforts should address depressive and anxiety symptoms during pregnancy together with equal importance, since the comorbidity increases the adverse birth and neonatal outcomes.

Maternal underweight is a well-established risk factor for LBW and PTB in low and lower middle-income countries [7, 35], as evident in our results from both east and west coasts. However, contrary to women in the east coast, physical IPV ever has emerged as a risk factor for LBW and PTB in the west coast. In the context of traditional gender role in Asian culture, women in Malaysia are primarily expected to care for children and manage household chores, irrespective of whether they are working outside home or not [36]. Women in west coast living in a contemporary culture are empowered, as evident by higher education, more women employed in service sectors and higher income, which may challenge the traditional views of gender role and social norms and elucidate the occurrence of IPV. Although research on the impact of IPV on birth outcomes has yielded mixed results, a sizable body of research have found IPV associated with LBW [37, 38]. IPV may impact negatively on maternal coping behaviour, such as smoking, alcohol and substance use, poor maternal nutrition, limited prenatal care, inadequate weight gain, and elevating stress, anxiety or depressive symptoms [39]. These elevated psychological stresses might exacerbate pre-existing conditions, such as hypertension, gestational diabetes, or it may lead to other pregnancy complications [40]. Stresses of experiencing IPV also alter the hypothalamic-pituitary-adrenal axis leading to changes in hormones that may affect the infants to be born with LBW and PTB [39], in the same way as depression and anxiety during pregnancy influence infants to born with LBW [1, 7].

ADS, but not AAS was independently associated with CS or instrumental delivery in the final model, but a significant association was found among women with ADS in west coast after stratification. The other risk factor of CS or instrumental delivery in the west coast was complications during delivery, which was also the strongest risk factor for CS and instrumental delivery in east coast, indicating that the obstetric interventions were mostly medically indicated. It is confirmed by Karalasingam et al. [22] who reported that CS was conducted in Malaysia due to obstetric indications, such as diabetes, hypertension and breech babies. The rate of CS is increasing globally, including in Malaysia (23%), despite WHO sets the target that CS rate should not exceed 15% in any population [22]. Research has shown mixed results about the association among ADS, AAS and CS, and most studies reported no association between mode of delivery and antepartum mental health [20, 41]. Our findings support the association between ADS at the third trimester of pregnancy and the risk for emergency CS [11], and ADS at 32 weeks of gestation and CS and instrumental delivery [42]. The mechanism by which ADS interferes with the mode of delivery may be due to negative influences of maternal mood on women’s confidence for delivery, which could contribute to fear of childbirth and lower pain threshold leading to prolonged labour and thus to increase prevalence of CS [43]. Our findings that women with ADS in the west coast were more likely to have CS is difficult to explain. Nonetheless the women with ADS experienced delivery complications, mainly the prolonged second stage of labour, which is one of the major causes for CS in west coast. Further research to explore the mechanism of how ADS impacts on prolonged labour is suggested. Moreover, women in west coast live in the most developed and modern part of Malaysia, with a life more alike with the western style and the prevalence of CS is always been high in western countries [11], depending towards more women preferring CS and, therefore, delivering by CS without medical indication [44].

The strengths of our study are that it includes a large number of pregnant women who were followed prospectively that helped us collect precise and reliable data. Our use of locally validated measures of ADS and AAS has increased validity of exposures. Although we adjusted for the effect of many priori confounders including IPV, the study has some limitations including an absence of the use of antidepressant medication during pregnancy [9] and lack of information on a number of variables such as anemia, hypertension, gestational diabetes and smoking (although smoking was uncommon among the women of our study population) [45]. Comorbidity was not considered in the results, which may confound the data as depression and anxiety often co-occur [34]. In addition, we have used categorical instead of continuous measures of ADS [7] and self-reported depression instead of clinical diagnosis, which could have increased the risk of PTB and LBW [33].

## Conclusion

In this study we found independent associations of ADS with newborn’s LBW, PTB and CS or instrumental delivery over and above the well-established risk factors of maternal underweight, low income and physical abuse ever, but not AAS. Taking into account the study sites, the association of ADS with LBW remains the same in both coasts, AAS becomes the predictor for both LBW and PTB in the east coast, and ADS for CS or instrumental delivery in the west coast. The clinical implication of this study is to integrate a universal screening intervention into antenatal care services in health clinics, where all pregnant women should be screened for ADS and AAS by the clinic nurses using locally validated EPDS and DASS-anxiety. Further, referring women with severe depressive and anxiety symptoms to the nearest psychiatric care facilities for accuracy of diagnosis and treatment of ADS and AAS combined with supporting women with IPV may help improve birth outcomes and reduce obstetric interventions.

## Data Availability

The dataset generated and analysed during the current study are not publicly available due to confidentiality issues but are available from the corresponding author on reasonable request.

## Abbreviations

AAS: Antepartum anxiety symptoms
ADS: Antepartum depressive symptoms
BMI: Body mass index
CS: Cesarean section
DASS: Depression, Anxiety and Stress Scale
EPDS: Edinburgh Postnatal Depressive Scale
GDP: Gross domestic product
IPV: Intimate partner violence
LBW: Low birth weight
MSPSS: Multidimensional Scale of Perceived Social Support
PTB: Preterm birth
SES: Socioeconomic status
